# Count regression models analysis of factors affecting antenatal care utilization in Ethiopia: original article

**DOI:** 10.1097/MS9.0000000000000705

**Published:** 2023-07-27

**Authors:** Birhanu Woldeyohannes, Zemene Yohannes, Habte T. Likassa, Gizachew G. Mekebo, Senahara K. Wake, Assefa L. Sisay, Ketema B. Gondol, Abebe Argaw, Gezahagn Diriba, Tariku Irana

**Affiliations:** a Technical and Financial Team Leader, Addis Ababa Transport Bureau, Addis Ababa, Ethiopia; b Assistant Researcher in Industrial Development Policy Studies Centre, Ethiopian Policy Studies Institute, Addis Ababa, Ethiopia; c College of Natural and Computational Science, Addis Ababa University, Addis Ababa, Ethiopia; d College of Natural and Computational Science, Ambo University, Ambo, Ethiopia; e Institute of Health Science, Jimma University, Jimma, Ethiopia

**Keywords:** antenatal care, count regression models, EMDHS 2019, factors affecting, ZIP model

## Abstract

**Background:**

Antenatal care (ANC) reduces maternal and newborn mortalities and makes it easier to find infections early and prevent them from progressing. This study aimed to identify factors affecting ANC utilization in Ethiopia via the count regression model.

**Methods:**

The data for the study was drawn from the 2019 Ethiopian Mini Demographic and Health Survey dataset. Count regression models, such as Poisson, Negative Binomial (NB), Zero-Inflated Poisson (ZIP), and Zero-Inflated Negative Binomial (ZINB) models, were used to determine the factors influencing ANC utilization.

**Results:**

A total of 3962 women were included in the study. Only about 42% of women used the WHO-recommended number of ANC of a minimum of four visits. The ZIP model was outperforming to fit the data as compared to other count regression models. Rural residence (IRR=0.8832, 95% CI: 0.8264–0.9440), being resident of the Somalia region (IRR=0.4762, 95% CI: 0.3919–0.5785), SNNPR (IRR=0.8716, 95% CI: 0.7913–0.9600), and Gambela Region (IRR=0.7830, 95% CI: 0.7063–0.8680), being Muslim (IRR=0.9384, 95% CI: 0.8876–0.9921) decrease the ANC utilization. Contrarily, Addis Ababa residence (IRR=1.1171, 95% CI: 1.0181–1.2259), primary education (IRR=1.1278, 95% CI: 1.0728–1.1855), secondary and higher education (IRR=1.2357, 95% CI: 1.1550–1.3220), middle wealth index (IRR=1.0855, 95% CI: 1.0095–1.1671) and rich wealth index (IRR=1.0941, 95% CI: 1.0152–1.1790) increase the ANC utilization.

**Conclusion:**

The ZIP model best fitted the data compared to others. The study revealed that being poor, rural resident, uneducated, Somalia region resident, SNNPR resident, Gambela region resident, and Muslim were factors associated with lower ANC utilization. Thus, health education is needed to be given for mothers with no education. In addition, building a strong awareness-creation program regarding ANC is required for rural residents so as to improve the level of ANC utilization in Ethiopia.

## Background

Highlights

- This study attempted to identify and describe factors affecting antenatal care (ANC) utilization in Ethiopia.
- The study was a secondary data analysis based on the 2019 Ethiopian Mini Demographic and Health Survey data.
- Count regression models were employed to identify factors affecting ANC utilization in Ethiopia and the ZIP model was outperforming to fit the data.
- The study found that place of residence, region, level of education, religion, and wealth index significantly affect ANC visits in Ethiopia.
- Health education is needed to be given for mothers with no education.

Antenatal care (ANC) is defined as the care provided to pregnant women and teenage girls by trained healthcare professionals to ensure the greatest possible health for both mother and unborn child throughout pregnancy^1^. Globally, quite a lot of mothers and newborns are at the risk of dying due to complications related to pregnancy and delivery of the baby^2^.

ANC reduces maternal and newborn mortalities and makes it easier to find infections early and prevent them from progressing^3,4^. About 86% of all maternal deaths worldwide occurred in the Sub-Saharan Africa and Southern Asia WHO regions in 2017; Sub-Saharan Africa accounted for almost 66% of these deaths^5^. Target 3.1 of the sustainable development goals (SDG) seeks to reduce maternal mortality rates worldwide to fewer than 70 per 100 000 live births by 2030^6^.

The WHO recommends a minimum of four ANC visits throughout pregnancy. Around 86% of pregnant women worldwide seek ANC at least once, but only 62% receive a minimum of four visits. Only 52% of pregnant women in sub-Saharan Africa go to at least four ANC visits^7^. Ethiopia, in Sub-Saharan Africa, is one of the nations with poor antenatal care utilization, with 74% of pregnant women receiving ANC and only 43% of pregnant women getting at least four ANC visits^8^.

ANC service utilization increases health facility delivery, which would reduce maternal morbidity and mortality^9,10^. Receiving ANC also increases the likelihood of mothers’ optimal feeding and exclusive breastfeeding of their infants^11–16^, which could prevent 823 000 under five deaths and 20 000 deaths from breast cancer annually^17^. In addition, ANC has a positive effect on the reduction of maternal and child mortality^18–25^. The ANC can also be a source of information for micronutrients, hypertension management to avoid tetanus vaccination, and prevention of HIV/AIDS transmission from mother to child^26–33^.

In Ethiopia, ANC visits by mothers from skilled providers increased from 28% in 2005 to 74% in 2019, with only 43% of women receiving them at least four times. The ANC coverage, in the country, from skilled providers is highest in the capital of the country, Addis Ababa (97%), while it is lowest in the Somali region (30%)^8^.

Several studies have shown ANC is affected by factors like place of residence, region, level of education, husband’s attitude towards ANC, level of education of husband, religion, marital status, ethnicity, frequency of reading newspapers, possessing radio, frequency of watching TV, monthly income, birth order, family size, knowledge about antenatal care, type of pregnancy, maternal age, parity, participation in decision making, average annual income, employment status, wealth index, and walking distance to/from a health facility^34–48^.

So as to meet the sustainable development goal of lessening the maternal mortality rate to fewer than 70 per 100 000 live births, neonatal mortality rate by fewer than 12 per 1000 live births, and under-5 mortality by fewer than 25 per 1000 live births by 2030^49^, encouraging mothers to attend antenatal care, along with other components such as skilled birth attendant delivery and family planning, is crucial as it plays a significant role in improving maternal and newborn health. Thus, this study aimed to identify factors influencing ANC utilization in Ethiopia based on the 2019 Ethiopian Mini Demographic and Health Survey (EMDHS) dataset using count regression models. The findings of this study will present important information on ANC and factors affecting the utilization of ANC services in the country.

## Methods

### Source of data

The data used for the analysis in this study were drawn from 2019 EMDHS data.

### Sample size

A sample of 3962 women aged 15–49 years was included in the study.

### Variables included in the study

The response variable was the total number of times of ANC visits a pregnant woman made in her recent pregnancy, and the independent variables were residence, region, age, family size, wealth index, educational level, marital status, religion, and birth order.

### Data analysis

The count regression models: Poisson, Negative Binomial (NB), Zero-inflated Poisson (ZIP), and Zero-inflated Negative Binomial (ZINB) were applied to identify factors affecting the number of ANC visits.

## Results

### Descriptive analysis results

In this study, of the total women included, 1044 (26.4%) had no ANC visit at all, and 141 (3.56%), 353 (8.91%), and 768 (19.37%) had ANC visits of one, two, and three times, respectively. About 42% attended an ANC visit at least four times and only 3% attended at least eight times. The maximum number of ANC visits made was 20 times, which was made by only three women. The mean number of ANC visits women made was 7.88, with a variance of 30.12. The variance (30.12) of the number of ANC visits was greater than the mean (7.88), indicating the possibility of over-dispersion (Table 1).

**Table 1:** Number of ANC visits made by mother during recent pregnancy in Ethiopia, 2019 EMDHS.

Number of ANC visits	Frequency	Percent
0	1044	26.35
1	141	3.56
2	353	8.91
3	768	19.37
4	847	21.37
5	393	9.92
6	196	4.95
7	100	2.52
8	64	1.62
9	26	0.66
10	18	0.45
11	3	0.08
12	4	0.10
13	1	0.03
15	1	0.03
20	3	0.08
Total	3962	100
Mean	7.88	
Variance	30.12	

ANC, antenatal care.

### Model selection

A model having the smallest Akaike Information Criterion (AIC) value and Bayesian Information Criterion (BIC) value is considered as best fit^50–52^. Accordingly, as the ZIP model has the minimum ACI and BIC, it best fits the data compared to others (Table 2).

**Table 2:** Model selection.

Model	AIC	BIC
Poisson	16 715.1	16 765.4
Negative Binomial (NB)	16 405.9	16 462.5
Zero-Inflated Poisson (ZIP)	14 658.5	14 897.3
Zero-inflated negative binomial (ZINB)	16 170.8	16 239.9

AIC, akaike information criterion; BIC, bayesian information criterion.

### Factors affecting ANC visits of mothers

The ZIP model, which was found to be the best model to fit the data, revealed that place of residence, region, educational level, religion, and wealth index were factors affecting ANC utilization. The number of ANC visits was 0.8832 (IRR=0.8832, 95% CI: 0.8264–0.9440) times lower among women residing in rural areas than those residing in urban areas. The number of ANC visits was 1.1171 (IRR=1.1171, 95% CI: 1.0181–1.2259) times higher among women living in Addis Ababa than in Tigray. Contrarily, the number of ANC visits were 0.4762 (IRR=0.4762, 95% CI: 0.3919–0.5785), 0.8716 (IRR=0.8716, 95% CI: 0.7913–0.9600), and 0.7830 (IRR=0.7830, 95% CI: 0.7063–0.8680) times lower among women living in the Somalia region, SNNPR, and Gambela region, respectively, than those living in the Tigray region. The number of ANC visits for mothers having primary education was increased by 12.78% (IRR=1.1278, 95% CI: 1.0728–1.1855) compared to uneducated ones. Similarly, the number of ANC visits for mothers having secondary and higher educational levels was increased by 23.57% (IRR=1.2357, 95% CI: 1.1550–1.3220) compared to uneducated mothers. The number of ANC visits for Muslim religion follower mothers was 0.9384 (IRR=0.9384, 95% CI: 0.8876–0.9921) times lower than for mothers who were Orthodox religion followers. The number of ANC visits for women with wealth indices of middle and rich were, respectively, 1.0855 (IRR=1.0855, 95% CI: 1.0095–1.1671) and 1.0941 (IRR=1.0941, 95% CI: 1.0152–1.1790) times lower than for poor mothers (Table 3).

**Table 3:** ZIP model results of factors affecting number of ANC visits in Ethiopia, 2019 EMDHS.

							95% CI for IRR	
Covariate	Category	Estimate	SE	*Z*	*P*	IRR	Lower	Upper
Intercept	Constant	0.9823	0.1536	6.39	0.000	2.6705	1.9762	3.6088
Residence (Ref: Urban)	Rural	−0.1242	0.0340	−3.66	0.000	0.8832	0.8264	0.9440
Region (Ref: Tigray)	Afar	−0.0766	0.0559	−1.37	0.070	0.9262	0.8301	1.0335
	Amahara	−0.0527	0.0401	−1.32	0.058	0.9487	0.8771	1.0262
	Oromia	−0.0625	0.0458	−1.36	0.072	0.9394	0.8587	1.0276
	Somali	−0.7420	0.0993	−7.47	0.000	0.4762	0.3919	0.5785
	Benishangul-Gumuz	0.0038	0.0443	0.08	0.094	1.0038	0.9203	1.0948
	SNNPR	−0.1375	0.0493	−2.79	0.005	0.8716	0.7913	0.9600
	Gambela	−0.2447	0.0526	−4.65	0.000	0.7830	0.7063	0.8680
	Harari	−0.0774	0.0490	−1.58	0.114	0.9255	0.8407	1.0188
	Addis Ababa	0.1108	0.0474	2.34	0.019	1.1171	1.0181	1.2259
	Dire Dawa	−0.0500	.4658	−1.07	0.082	0.9513	0.8683	1.0422
Age (Ref: <25)	25–34	−0.1014	0.0602	−1.96	0.801	0.9001	0.7860	1.109
	35 and older	−0.0803	0.1213	−0.65	0.600	0.9303	0.7205	1.124
Educational of level (Ref: no education)	Primary	0.1202	0.0255	4.72	0.000	1.1278	1.0728	1.1855
	Secondary and higher	0.2116	0.0345	6.14	0.000	1.2357	1.1550	1.3220
Birth order (Ref: First)	2nd–4th	−0.1403	0.088	−1.47	0.2006	0.8651	0.7053	1.0293
	5th and above	−0.0870	0.079	−1.10	0.152	0.9072	0.7561	1.0574
Religion (Ref: Orthodox)	Catholic	−0.2440	0.1535	−1.59	0.082	0.7835	0.0800	1.0584
	Protestant	−0.0805	0.0359	−2.24	0.069	.9227	0.8600	1.008
	Muslim	−0.0636	0.0284	−2.24	0.025	0.9384	0.8876	0.9921
	Traditional	−0.2311	0.1818	−1.27	0.104	0.7937	0.5557	1.1336
	Other	−2602	0.2283	−1.14	0.054	0.7709	0.4928	1.2767
Wealth index (Ref: Poor)	Middle	0.0820	0.0370	2.22	0.027	1.0855	1.0095	1.1671
	Rich	0.0899	0.0382	2.36	0.018	1.0941	1.0152	1.1790
Family size (Ref: <5)	5 and more	−0.0480	0.1271	−0.40	0.581	0.9460	0.7081	1.1850
Marital status (Ref: Never in union)	Married	0.1768	0.1378	1.28	0.099	1.1934	0.9109	1.5633
	Living with partner	0.2249	0.1709	1.32	0.088	1.2522	0.8958	1.7506
	Widowed	0.1840	0.1723	1.07	0.086	1.2020	0.8755	1.6849
	Divorced	0.1811	0.1461	1.24	0.078	1.1985	0.9001	1.5959
	Separated	0.1154	0.1557	0.74	0.059	1.1223	0.8272	1.5227

IRR, Incidence Rate Ratio; Ref, Reference Category.

## Discussion

About 26.4% of the women, included in this study, did not attend an ANC visit while the remaining 73.6% attended an ANC visit minimum of once in their recent pregnancy. The ZIP model revealed that place of residence, educational level, religion, region, and wealth index were significantly affecting ANC utilization in Ethiopia.

In this study, the region was found to be significantly associated with ANC utilization services. Women living in Addis Ababa’s city administration were more likely to utilize ANC service compared to those living in Tigray. This might be due to the fact that Addis Ababa has better healthcare options and living conditions than other areas. Contrarily, women living in the Somalia region, SNNPR, and Gambela region were less likely to utilize ANC service compared to those residing in the Tigray region. It was also revealed that rural women were less likely to utilize ANC services compared to urban women. This is supported by the studies^35,42,44,53,54^. This might be due to the reduced availability of educational services and knowledge about ANC services in rural areas.

This study also showed that the educational level of the mother had a favorable link with the utilization of ANC and that ANC visits rise as the mother’s educational level rises. This result agrees with the results of the prior studies^35,42,44,48,54–56^. This might be due to the fact that better education can help them have a better understanding and awareness regarding ANC services.

It was also found that religion had a significant association with the utilization of ANC service. Muslim religion followers mothers were less likely to utilize the ANC service compared to Orthodox religion followers. Furthermore, the ANC utilization was found to be significantly affected by the wealth index. Women with middle and rich wealth indices were more likely to use ANC services than women with poor wealth indices. This finding is consistent with the research findings of studies^35,42,54,57^.

## Conclusion

This study aimed mainly to identify factors affecting ANC utilization in Ethiopia via a count regression model. The ZIP model outperformed to fit the data as compared to other count regression models. Place of residence, region, educational level, religion, and wealth index were significantly influencing factors of the utilization of ANC in Ethiopia. Thus, health education is needed to be given to mothers with no education. In addition, building a strong awareness-creation program regarding ANC is required for rural residents so as to improve the level of ANC utilization in Ethiopia.

## Ethical approval

The data used for this study was secondary data and publicly available and has no personal identifiers.

## Consent

None.

## Sources of funding

None.

## Author contributions

Z.Y.: conceptualization, data curation, formal analysis, investigation, methodology, software, validation, and writing – original draft; B.W.: data curation, formal analysis, investigation, methodology, software, validation, and writing – review and editing; H.T.L.: formal analysis, investigation, methodology, software, validation, and writing – review and editing; G.G.M.: formal analysis, investigation, methodology, software, validation, and writing – review and editing; S.K.W.: formal analysis, investigation, methodology, software, and writing – review and editing; A.L.S.: formal analysis, methodology, software, and writing – review and editing; K.B.G.: formal analysis, methodology, software, and writing – review and editing; A.A.: formal analysis, methodology, software, and writing – review and editing; G.D.: formal analysis, methodology, software, writing – review and editing; T.I.: formal analysis, methodology, software, writing – review and editing.

## Conflicts of interest disclosure

The authors declare that they have no conflicts of interest.

## Provenance and peer review

Not commissioned, externally peer reviewed.

## Data availability statement

The data used for this study is available upon reasonable request from the corresponding author.
